# Pollen image manipulation and projection using latent space

**DOI:** 10.3389/fpls.2025.1539128

**Published:** 2025-02-28

**Authors:** Ben Mills, Michalis N. Zervas, James A. Grant-Jacob

**Affiliations:** Optoelectronics Research Centre, University of Southampton, Southampton, United Kingdom

**Keywords:** pollen, latent space, deep learning, evolution, imaging

## Abstract

Understanding the structure of pollen grains is crucial for the identification of plant taxa and the understanding of plant evolution. We employ a deep learning technique known as style transfer to investigate the manipulation of microscope images of these pollens to change the size and shape of pollen grain images. This methodology unveils the potential to identify distinctive structural features of pollen grains and decipher correlations, whilst the ability to generate images of pollen can enhance our capacity to analyse a larger variety of pollen types, thereby broadening our understanding of plant ecology. This could potentially lead to advancements in fields such as agriculture, botany, and climate science.

## 1 Introduction

Pollen grains are essentially the male gametes of plants, carrying the necessary genetic material for plant reproduction (Knox et al., 1986). The role of pollen in plants is crucial, as it allows plants to reproduce without relying on water for the transport of biological components necessary for fertilisation. Pollen grains come in a range of sizes and substructures at the nanometre scale (Halbritter et al., 2018). The morphology of these pollen grains such as shape, ornamentation, and aperturation (Mert, 2009) play a crucial role in processes like germination (Matamoro-Vidal et al., 2016). The surface of pollen grains can have unique features that help them cling to different modes of transportation, such as bird feathers, bee legs, or animal fur, or help them sail through the air on appendages that resemble airplane wings or hot air balloons. A pollen grain’s morphology can change due to dehydration (Fatmi et al., 2020), as dehydration can cause pollen to become more angular or irregular as the turgor pressure that maintains its shape is lost. Therefore, imaging of pollen grains is a crucial technique, as it provides information on the pollen’s morphology in 2D and 3D, providing key insights into the health of crops and the environment (Lau et al., 1995; Fernandez-Mensaque et al., 1998). Various imaging methods, including fluorescence microscopy (Atlagić et al., 2012), electron microscopy (Coutinho and Dinis, 2009), and X-ray tomography (Wang et al., 2015; Li et al., 2016) have been used to discern the external and internal structures of pollen grains. Despite their capabilities, these techniques have limitations. Fluorescence microscopy relies on specific staining protocols that can obscure natural morphological details and require precise sample preparation. Electron microscopy, although capable of high-resolution imaging, is limited to surface morphology and necessitates labour-intensive preparation steps; and whilst X-ray tomography offers 3D imaging, it is resource-intensive, involving costly equipment and time-consuming data analysis. Additionally, these methods are unsuitable for high-throughput analysis due to the extensive time and expertise required, making them impractical for studying the vast diversity of pollen species on a large scale.

Analytical methods have also been employed to explore the creation of pollen grain apertures, such as the work by Zhou and Dobritsa (2019), which used genetic and molecular biology approaches to investigate the regulatory pathways controlling aperture formation in pollen grains. Their study focused on the role of specific proteins and genes in determining the placement and structure of apertures, which are critical for pollen function and viability. Whilst this research provides fundamental insights into pollen development, it relies on labour-intensive experimental techniques and lacks scalability for analysing large numbers of pollen species.

Owing to the vast number of pollen species, additional methods of pollen analysis and identification have been sought to help understand pollen and thus plant ecology.

Over the past 10 years, advancements in graphics processing units (GPUs) and deep learning algorithms have ushered in a new era of large-scale, data-driven research (LeCun et al., 2015). The convolutional neural network (CNN), which is inspired by the visual cortex (Serre et al., 2007), can be used to categorise images by outputting a label or value. CNNs have been applied across the field of palynology (Daood et al., 2016; Grant-Jacob and Mills, 2022; Romero et al., 2020; Punyasena et al., 2012), with examples including pollen identification via visible light microscopy of pollen grain types (Mahbod et al., 2021; Crouzy et al., 2016; Marcos et al., 2015; Grant-Jacob et al., 2021), identification of 46 different pollen grain types (Sevillano et al., 2020), and identification of pollen grains from scattering (Grant-Jacob et al., 2019, 2018) and holographic patterns (Sauvageat et al., 2020; Luo et al., 2022).

In recent years, with the application of deep learning models, CNNs have shown great promise in the field of palynology for pollen grain classification and analysis. However, existing studies primarily focus on classification tasks using real pollen images, often limited by the scarcity of high-quality and diverse datasets. Whilst CNN-based models have demonstrated impressive results in classifying pollen grains, such as the POLEN23E dataset for 23 pollen types with over 97% accuracy (Sevillano and Aznarte, 2018) and the classification of 73 different pollen types with a higher than 90% accuracy (Astolfi et al., 2020) using the POLLEN73S dataset that comprises 2,523 images, these methods face challenges when dealing with underrepresented species or rare morphological features.

These papers highlight the potential of deep learning in analysing and classifying pollen grains, which can significantly contribute to a range of fields such as agriculture, botany, and climate science. By automating the process of pollen identification, it becomes possible to analyse a larger variety of pollen types, thereby broadening our understanding of plant evolution and ecology.

Style Generative Adversarial Network (StyleGAN) (Karras et al., 2020), first introduced by NVIDIA researchers in 2018, is a type of generative neural network that has brought significant modifications to the generator model by using an alternative generator architecture that is borrowed from the style transfer literature, which allows it to create and subsequently modify synthetic (generated) images.

StyleGAN is potentially more effective than other generative models in producing realistic images because it introduces a unique style-based generator architecture that allows precise control over image attributes at different levels of abstraction, from coarse to fine details. Additionally, its disentangled latent space enables the generation of high-quality, diverse, and realistic images with smooth interpolation across variations. This makes it an ideal tool for creating synthetic pollen images that retain realistic qualities, which is vital for training deep learning models or enhancing datasets in fields such as agriculture, botany, and climate science.

The unique feature of such a style-based network is its ability to control specific aspects of the generated image through the manipulation of the latent space, which describes a higher-abstraction representation of the generated image. This allows for the generation of images with specific characteristics, such as a particular style or feature. For instance, in the case of generating images of faces, the network can control aspects such as the facial expression, identity, and even details like freckles or hair.

Previous synthetic generation studies in palynology have focused on generating low-resolution microscope images (Khanzhina et al., 2022) or using scanning electron microscope (SEM) images for higher-resolution representations (Grant-Jacob et al., 2022b). Whilst these approaches have been useful for generating synthetic data, they do not demonstrate the potential of using StyleGAN for offering new insights into the relationship between these traits using multiple vector manipulation, nor do they project real images into latent space for manipulation.

Unlike traditional approaches, this study introduces a novel application of StyleGAN for the synthetic generation of pollen images, enabling the manipulation of multiple latent space vectors to control specific morphological features such as size, shape, and ornamentation to generate new images of pollen. Furthermore, it allows for the exploration of feature relationships, such as the correlation between size and ornamentation, which is often difficult to achieve through conventional imaging techniques.

This paper explores the potential of using StyleGAN for interpolating between microscope images of pollen grains in latent w-space to generate additional images of specific pollen taxa and to simulate transformations from one pollen taxon to another. It also demonstrates that w-space latent vectors can be identified that allow characteristics, such as pollen size and shape, to be manipulated in generated images and that this technique could potentially unlock further understanding of the palynological relationships.

**Figure 1:**
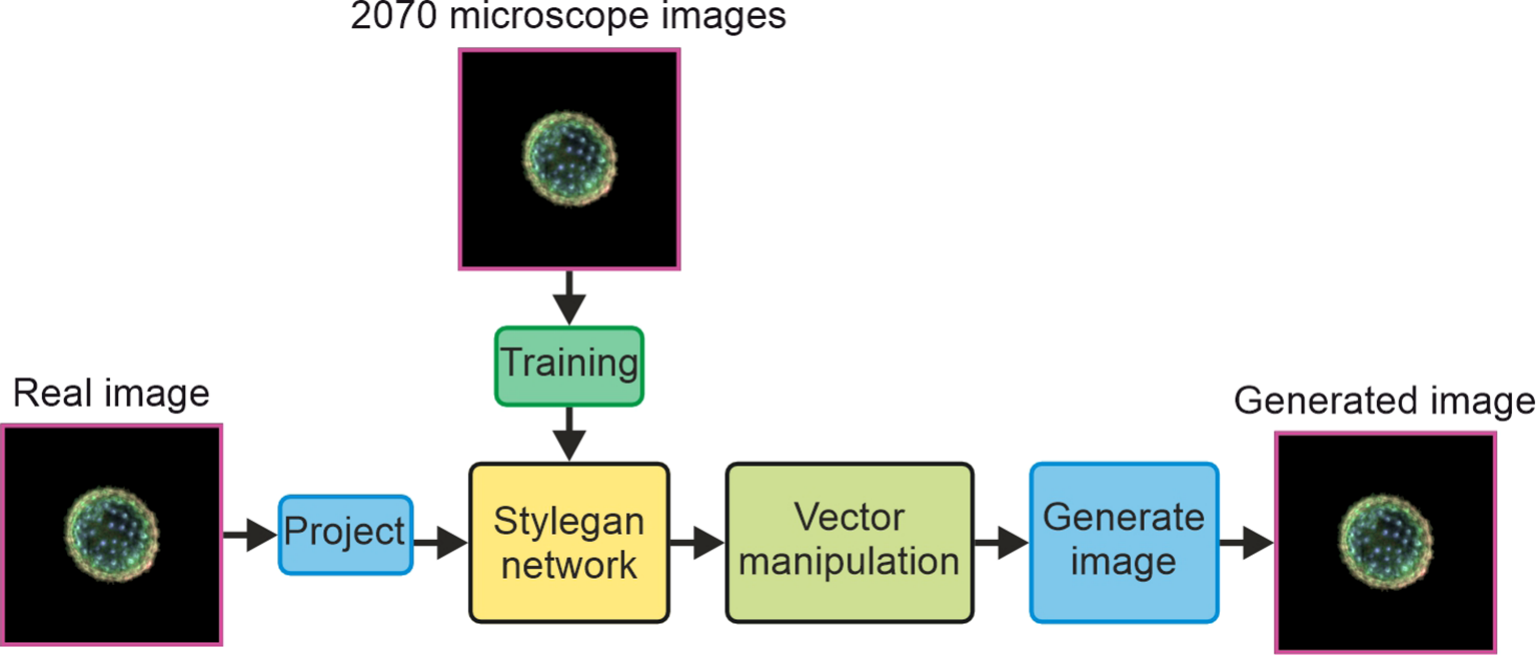
Block diagram concept of the study.

A block diagram concept of the study demonstrated in this manuscript is displayed in **Figure 1**, showing how 2,070 microscope images are used to train a StyleGAN network, and then a single real image is projected into the network and undergoes vector manipulation in latent space before being generated by the synthesis part of the StyleGAN network.

## 2 Materials and methods

### 2.1 Data acquisition

The image data used in this work were derived from the pollen dataset published by Sevillano et al. (2020) and further detailed in Holt (2020). This dataset comprises high-resolution microscope images of 46 different pollen taxa. The dataset is particularly well-suited for this research due to its diversity of taxa, which spans a wide range of morphological characteristics such as size, shape, and ornamentation. These variations are critical for training machine learning models to recognise and classify different pollen types accurately.

To make sure that there was equal weighting in the training of the neural network, 45 images of each pollen taxon were used in training the neural networks (2,070 total). The image files from the dataset were padded with zeros and then resized to 256 × 256 pixels (RGB), to ensure that all images had the same aspect ratio whilst preserving the relative size information, before being used as training data.

### 2.2 Neural networks

This work used two separate neural networks: the StyleGAN network and the CNN. The StyleGAN network was used to generate images of pollen and subsequently modify specific properties of these generated images. Owing to a CNN’s ability to classify pollen grains with great accuracy, a CNN was used to validate the generated images. The CNN was used as a classifier network to identify the taxa of each image generated by the StyleGAN network. The neural networks underwent training on a workstation running Windows 10 and equipped with an AMD Ryzen Threadripper PRO 5975WX with 32 cores operating at 3.60 GHz, 128 GB RAM, and two NVIDIA A6000 GPUs (each with 48 GB memory).

**Figure 2:**
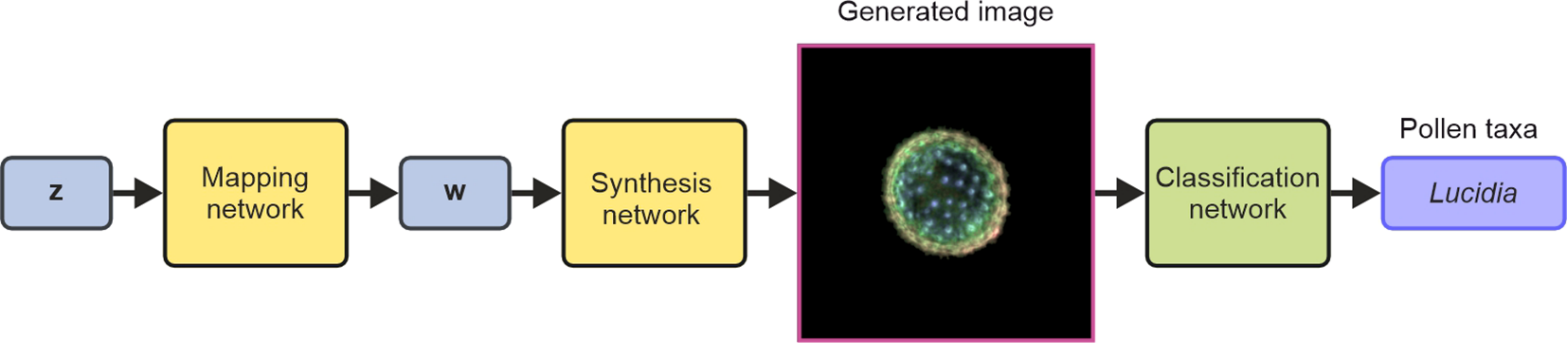
Schematic of the StyleGAN neural network (formed of the mapping and synthesis networks) for generating images and the use of a CNN for subsequent classification of the generated images. StyleGAN, Style Generative Adversarial Network; CNN, convolutional neural network.

StyleGAN, a generative neural network, was used to create synthetic microscope images of pollen grains, where the appearance of these images was based on the training data. This work used StyleGAN2, which is available on GitHub (https://github.com/NVlabs/stylegan2-ada-pytorch.git). As shown in **Figure 2**, the StyleGAN network consists of two subnetworks known as the mapping network and the synthesis network. The mapping network transformed a random noise vector **z** (1 × 512) into a vector **w** (1 × 512), and the synthesis network transformed the vector **w** into an RGB image (256 × 256 × 3) of a pollen grain. Therefore, either a **z** or **w** vector could be used to generate a synthetic image of a pollen grain. The **z** vector is known as a latent space vector in z-space, and likewise, the **w** vector exists in w-space. Critically, as the mapping network is designed to disentangle the properties (or “style”) of pollen grains in the generated images, vectors in w-space correspond to a higher abstraction of the pollen grains and hence offer the capability to unlock manipulations of specific features in the generated images. In this work, as discussed later, this capability enables properties such as the size or shape of pollen grains in the generated images to be modified or for an image of a generated pollen grain to be gradually transformed into an image of a different pollen grain.

**Figure 3:**
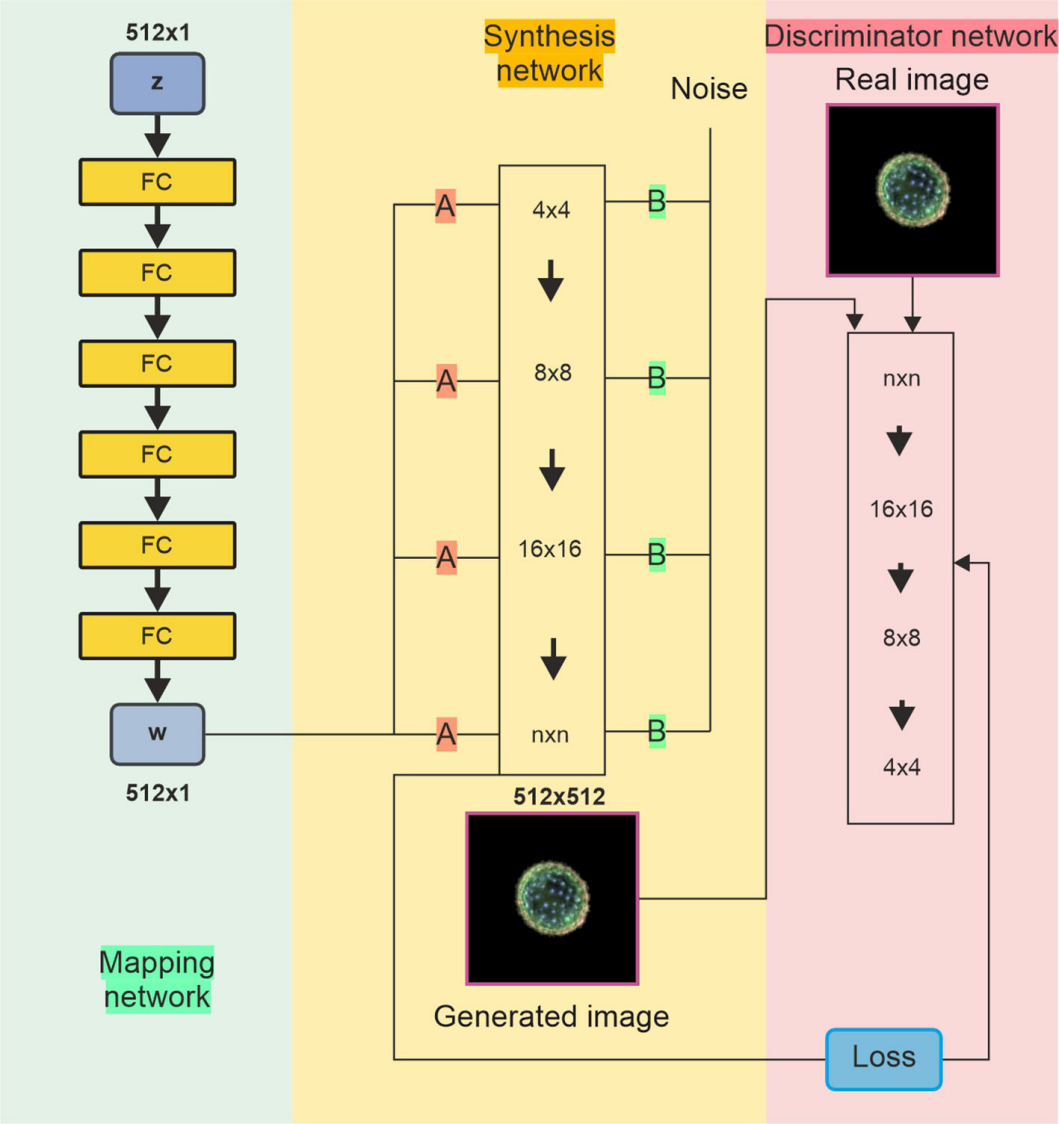
Diagram of StyleGAN with three networks. (Left) The mapping network transforms a random input into a style signal, controlling various aspects of image generation. (Middle) The synthesis (generator) network uses the information **(A)** from the mapping network to generate images from low to high resolution. It also incorporates random noise **(B)** to introduce variations and fine details. (Right) The discriminator network compares real and generated images, updating the weights of all networks through adversarial training to enhance performance. StyleGAN, Style Generative Adversarial Network.

The architecture of the StyleGAN network is shown in **Figure 3**. The StyleGAN architecture consists of three main components: the mapping network, synthesis network, and discriminator network. The mapping network (left panel) consists of fully connected layers and takes a latent vector (z) sampled from a distribution (e.g., Gaussian noise) and transforms it into an intermediate latent space (w), which helps disentangle features for better control during image generation. The synthesis network (middle panel) starts with a learned constant tensor at a low resolution (4 × 4) and progressively increases the resolution through up-sampling (e.g., 4 × 4 → 8 × 8 → 16 × 16) up to the final resolution (n × n). Noise injections at each level add stochastic details, resulting in a synthesised image. The discriminator (right panel) processes real or generated images, progressively reducing their resolution from high (n × n) to low (4 × 4) and outputs a prediction to differentiate between real and generated images. This network is connected to a loss function used to train both the generator and discriminator adversarially, with the generator aiming to produce images indistinguishable from real ones, whilst the discriminator tries to correctly classify them as real or fake.

The training for the StyleGAN was conducted using Python with CUDA enabled. The network was trained on 2,070 images with 5,000 kimg (5 million images processed), meaning that each of the training images was used approximately 2,400 times. The training process took approximately 2 days 18 hours, averaging 47 seconds per kimg. The training parameters included a learning rate of 0.0025, a non-saturating logistic loss function, and a batch size of 32, and Adaptive Moment Estimation (ADAM) was used as the optimiser (Kingma and Ba, 2014). The Fréchet inception distance (FID) score (Heusel et al., 2017) was used to measure the similarity between the generated images and the training images. This score represents the distance between the feature vectors of the two sets of images, where a score of zero would mean that the distributions of the generated and training images are identical. The FID score was computed every 200 kimg during the training process, and the score was observed until it plateaued around a value of 29 after 5,000 kimg.

**Figure 4:**
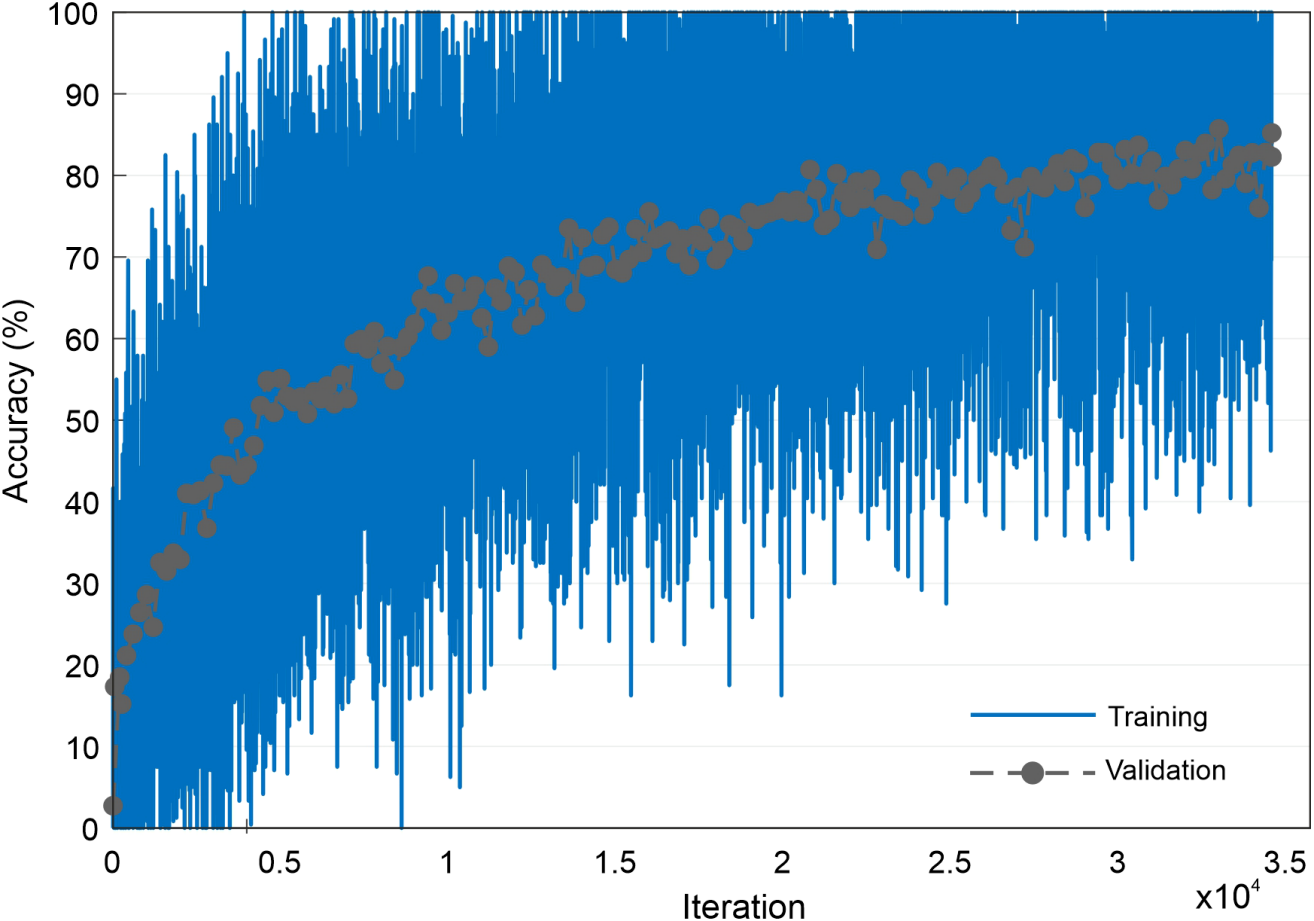
Graph showing the accuracy of training and validation progress during training of the CNN. CNN, convolutional neural network.

The CNN was trained to identify the taxa of the images generated by the StyleGAN neural network and was trained using the same image data as the StyleGAN network. Therefore, an image of 256 × 256 × 3 size was used as the input to the CNN, and the network output was a prediction of the pollen taxa. There were no other pre-processing steps beyond resizing and cropping. The training data for the CNN were split into percentages of 70% for training, 25% for validation, and 5% for testing, and the architecture was the Inception v3 (Szegedy et al., 2017) used in MATLAB (https://uk.mathworks.com/help/deeplearning/ref/inceptionv3.html, https://uk.mathworks.com/matlabcentral/fileexchange/65679-deep-learning-toolbox-model-for-inception-v3-network). In this work, no augmentation was used on the dataset. The network was trained for 5 epochs, with an initial learning rate of 0.0002, a validation frequency of 200, a learn rate drop factor of 0.1, and a minibatch size of 2; it took 2 days 21 hours 48 minutes to train. The CNN achieved a classification accuracy of 86% [see **Figure 4** for training accuracy and validation accuracy graph over 5 epochs (34,580 iterations) when applied to the testing data and is labelled as the classification network in **Figure 2**].

### 2.3 Image generation

**Figure 5:**
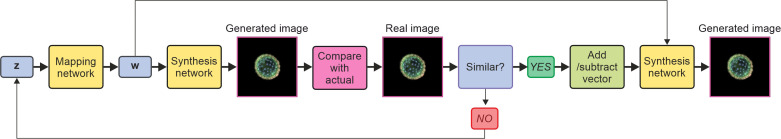
Schematic of methodology of projecting an image into latent space by generating random z vector, generating an image, and then comparing that image with the projected to obtain the suitable vector in latent space. The vector is then manipulated by adding or subtracting a vector before the synthesis network generates a new image.

**Figure 5** shows a schematic of the methodology for projecting an image into latent space and then extracting the relevant vector before manipulating the vector in latent space, either to increase or decrease the size of the pollen or to increase or decrease the spikiness. Compared to other studies on pollen image generation, we demonstrate the manipulation of latent space for image manipulation in multiple ways, such as size and shape, and we also project experimentally obtained images into latent space and manipulate them, potentially opening the opportunity to manipulate images and thus species not present in the training data.

We generated 1,000 synthetic images of pollen grains using 1,000 z vectors, where each of the 512 numbers in the vectors was randomly sampled from a normal distribution with µ = 0 and σ = 1. The corresponding w-vectors for each of these generated images were also recorded to support subsequent latent space manipulations. Interpolation between two generated images with w-space vectors of wa and wb could then be achieved by generating an image using a w-space vector of w_c_ = w_a_ + k(w_b_ − w_a_), where k is a scalar between 0 and 1 and wc is the w-vector for the interpolated image. In this case, the vector (w_b_ − w_a_) therefore corresponds to a w-space vector that describes the structural change between the two images. If, for example, wa corresponded to a small pollen grain and wb corresponded to a large pollen grain, then (w_b_ − w_a_) would correspond to a w-space vector for increasing the size of the pollen grain. However, this w-space vector would also correspond to the changes in other features, such as the difference in the shape of the two pollen grains. Therefore, by averaging over many such vectors, a vector for increasing pollen grain size was identified that encapsulates the visual information contained in the training dataset. This “size” w-space vector could then be added to (or subtracted from) any w-space vector corresponding to a generated image to increase (or decrease) the size of the pollen grain in the generated image. To achieve this, a folder of synthetic images of “small” pollen grains was created, and a folder of “large” pollen grains was created, from which the w-space vectors were obtained. Similarly, a “spike” w-space vector for pollen transitioning from no spikes to spikes and a “round” w-space vector for pollen transitioning from triangular to round were identified. As shown later in this work, this allows the generation of a wide variety of images representing different types and sizes of pollen grains, along with bespoke morphological changes to the pollen grains in these images.

## 3 Results

Following the training of all the two neural networks, 1,000 z-space vectors were used to create 1,000 images using the StyleGAN neural network, and the corresponding 1,000 w-space vectors were also recorded. The CNN was then used to predict the taxa for each of these generated pollen grains.

**Figure 6:**
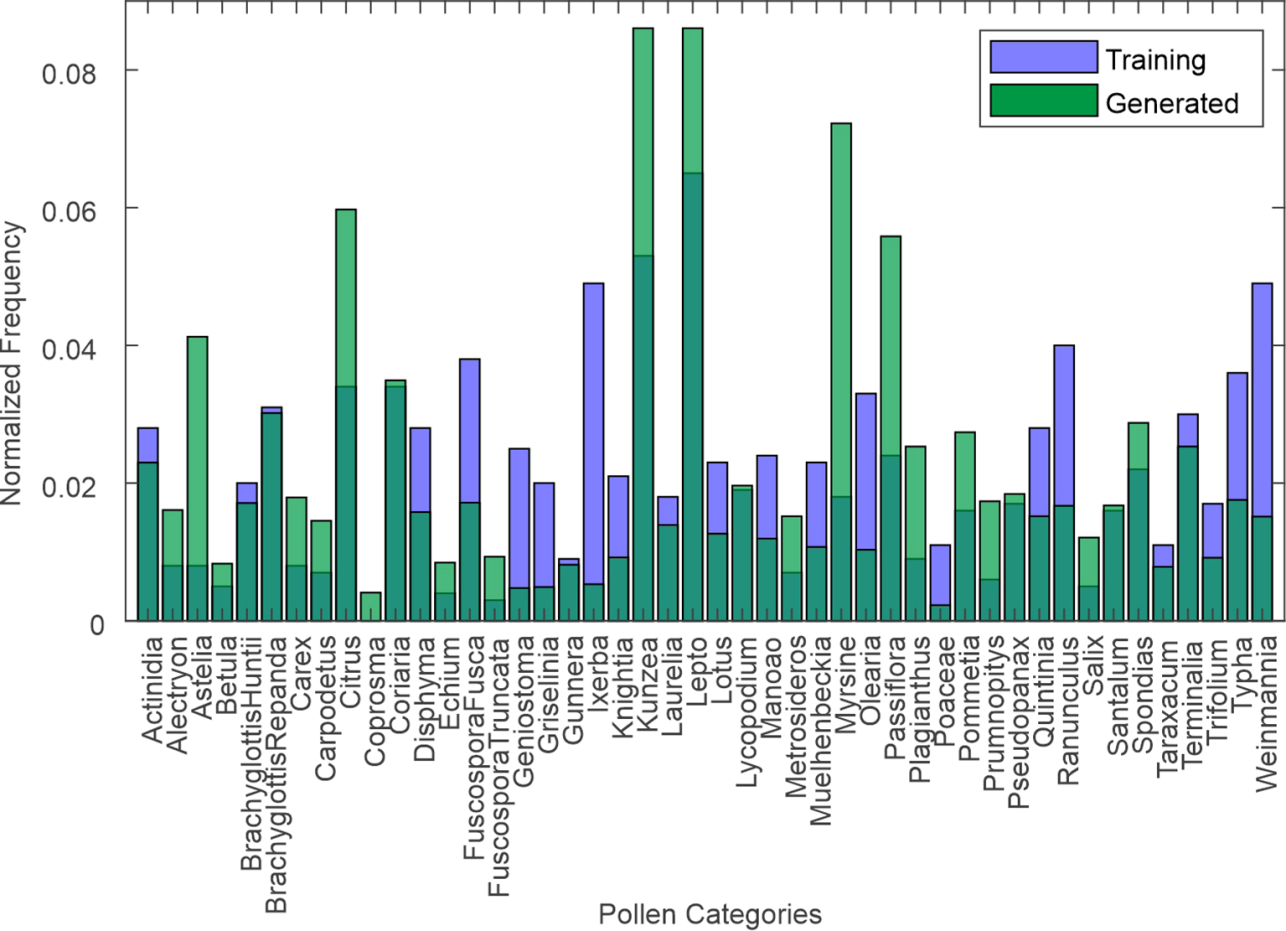
Histogram of distribution of taxa in training dataset and generated dataset (as predicted by CNN). CNN, convolutional neural network.

The selection of 1,000 z-space vectors was guided by the need to balance computational feasibility with adequate coverage of the latent space’s variability. The latent space of StyleGAN is inherently high-dimensional, and z-space vectors are typically modelled as a standard Gaussian distribution, meaning that random sampling spans a representative subset of the space. Generating 1,000 synthetic images is a practical compromise, manageable in terms of resources whilst sufficiently capturing variability for downstream tasks. To ensure that the generated images reflected the training dataset’s diversity, the FID was used for validation, giving a value of 6.281 for 5,000 kimg. Lower scores indicate higher similarity between the feature distributions of the real and synthetic datasets. Scores below 10 are considered very good in most generative model tasks, indicating higher similarity between the feature distributions of the experimental training and generated datasets. In addition, we compared the distribution of taxon labels in the training data with the 1,000 predicted labels of the generated data, as shown in **Figure 6**.

The Jensen–Shannon (JS) divergence (Nielsen, 2019) is a symmetric and bounded metric that measures the similarity between two probability distributions, making it interpretable and robust. A value of 0 indicates identical distributions, and the low divergence observed here suggests that the synthetic data closely approximate the real dataset, capturing much of its variability with only minor differences.

The JS divergence is calculated as


$$D_{JS}(P||Q)=\frac{1}{2}D_{KL}(P||M)+\;\frac{1}{2}D_{KL}(Q||M)$$

where *P* and *Q* are the two distributions being compared, *M* = 1/2(*P* + *Q*) is the average distribution, and *D_KL_* is the Kullback–Leibler divergence. The JS divergence is bounded between 0 and 1 [or log(2)) for certain bases] and symmetrically averages over both distributions. The JS divergence was calculated to be 0.073. This, along with the histogram comparison, indicates a relatively low divergence between the real and synthetic datasets.

The low JS divergence highlights that the synthetic dataset does an accurate job of replicating the variability of the real dataset. However, the histogram reveals that some specific categories may still benefit from refinement.

**Figure 7:**
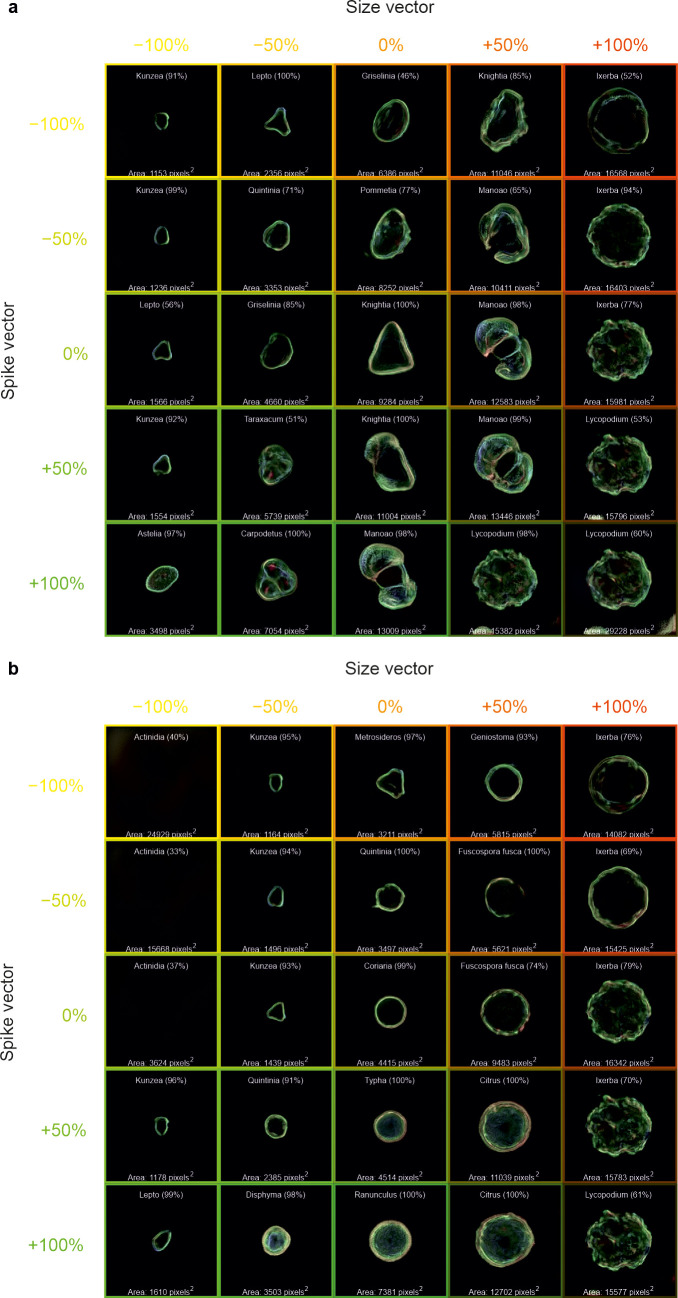
Generated images of pollen grains created through latent w-space vector manipulation, showing the addition of a “size” vector (−100%, −50%, 0%, +50%, and +100%) in the horizontal direction and a “spike” vector (−100%, −50%, 0%, +50%, and +100%) in the vertical direction, to generated images of **(a)** *Knightia* and **(b)** *Coriaria*. Each generated image also shows the predicted pollen taxa and predicted confidence, as well as the pollen size in pixels. Labelling is omitted from images without any visible grains.

The calculated w-space vectors of “size”, “spike”, and “round” were added (or subtracted) from a range of generated images to visualise the predicted changes in the morphology of the generated pollen. As displayed in **Figure 7**, −100%, −50%, 0%, +50%, and +100% of the “size” and “spike” vectors were added onto the w-space vector corresponding to a generated image of a) *Knightia* and b) *Coriaria*. The central image shows the generated images with no additional w-space vectors; the change in the horizontal direction shows the addition (or subtraction) of the “size” vector, and the change in the vertical direction shows the addition (or subtraction) of the “spike” vector. The classification CNN was applied to these generated images, and the predicted pollen taxa, along with prediction confidence, are shown on each generated image. Each generated image also includes the area of the pollen grain, calculated by summing the number of image pixels corresponding to the pollen grain. However, labelling is omitted from images without any visible grains.

**Figure 8:**
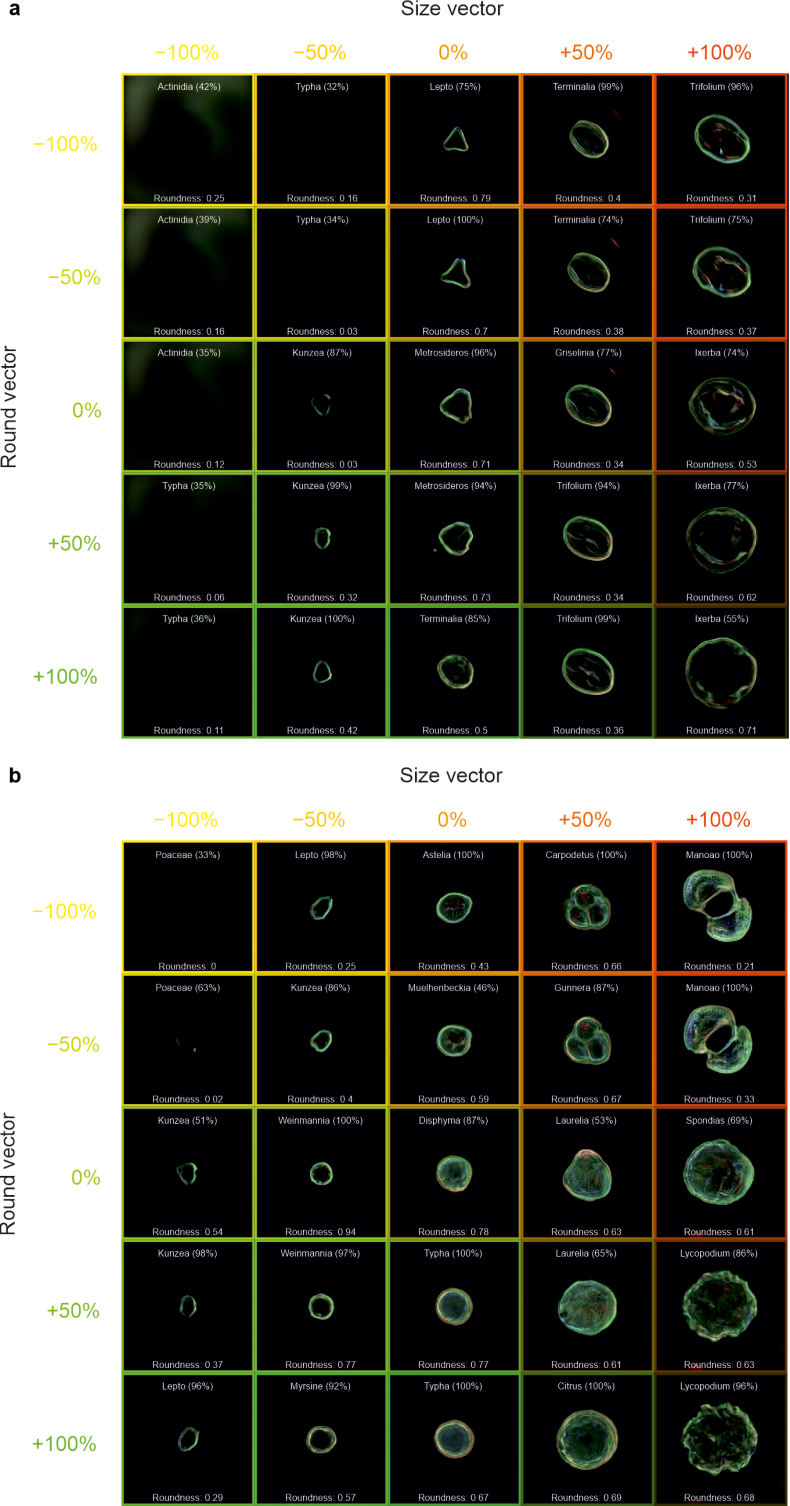
Generated images of pollen grains created through latent w-space vector manipulation, showing the addition of a “size” vector (−100%, −50%, 0%, +50%, and +100%) in the horizontal direction and a “round” vector (−100%, −50%, 0%, +50%, and +100%) in the vertical direction, to generated images of **(a)** *Metrosideros* and **(b)** *Disphyma*. Each generated image also shows the predicted pollen taxa and predicted confidence, as well as the circularity of the pollen grain. Labelling is omitted from images without any visible grains.

In **Figure 8**, the same methodology was also applied to generated images of a) *Metrosideros* and b) *Disphyma*, with the “size” vector applied in the horizontal direction and the “round” vector applied in the vertical direction. The roundness value is also labelled in the figure, which quantifies how closely the shape resembles a perfect circle, with higher roundness values indicating shapes that are more circular and lower values corresponding to more irregular shapes, where this value was determined as [(4πArea/Perimeter^2^) × (1 − 0.5/r)^2^, where r = Perimeter/(2π) + 0.5].

**Figure 9:**
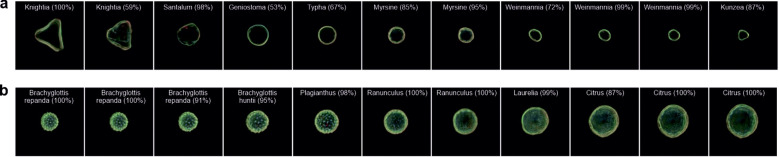
Generated images of pollen grains created through latent w-space vector manipulation, showing the interpolation of projected images between **(a)** *Knightia* and *Kunzea* and **(b)** *Brachyglottis repanda* and *Citrus*. Each generated image also shows the predicted pollen taxa and predicted confidence.

The ability to use images of the real world for interpolation could allow previously unseen pollen grains to be examined. As such, by projecting an experimental image into the latent space, an equivalent latent space image can be found and used in vector manipulation. The process of mapping a real-world image into the latent space of the model is known as “projection”. An initial latent vector is created, usually either at random or based on the average latent vector of the model. This latent vector is then progressively refined using gradient descent to reduce the disparity between the image produced from the latent vector and the original real-world image. The outcome of this iterative optimisation is a fine-tuned latent vector that encapsulates the real-world image in the model’s latent space. This vector can be further manipulated or analysed as needed. Overall, this procedure enables the model to effectively translate real-world images into its own latent space. In **Figure 9**, we interpolate between two different taxa in a) *Knightia* (LHS) and *Kunzea* (RHS) and b) *Brachyglottis repanda* (LHS) and *Citrus* (RHS) to demonstrate the capability of such a technique. We also use the CNN to predict the generated pollen taxa. As seen in the images, it is possible to interpolate between two taxa, generating what appear to be other taxa in the process.

## 4 Discussion

The generated images shown in **Figure 7** demonstrate that when the “spike” vector is added (or subtracted), not only does the appearance of the spikes on the generated pollen grains change, but the taxa also change. Likewise, as the “size” vector is added (or subtracted), the generated pollen changes size and taxa. When the size or spike vector is too negative, there is no image generation, meaning that the latent space vectors may not be mapped to the features that generate an image of pollen. When both vectors are applied, such as +100% “size” and +100% “spike”, a generated image that resembles *Lycopodium* pollen is created. It should be noted that whilst the CNN predicts *Lycopodium*, it is limited to the dataset on which it has been trained, and the actual *Lycopodium* pollen contains more ornamentation on its surface. Interestingly overall, when the “spike” is added, the size of the pollen grain increases, perhaps implying a correlation between size and spikes (or ornamentation), based on the dataset and calculated vectors used in this work. Indeed, this can be observed in **Figures 7A, B**, where only the edge of the pollen grains was visible in the smaller grains, and as the spike vector is increased, the pollen changes taxa to those that are not only larger but also have more ornamentation over the whole pollen.

The ability to manipulate images in latent space is also demonstrated by changing the roundness in **Figure 8**. It can be seen that the “size” vector increases the size of the generated pollen grain and that the “round” vector generally increases the circularity. In **Figure 8A**, there is little evidence that the roundness of the pollen has a correlation with the size, although for some taxa [e.g., *Griselinia* (0%, 50%) and *Ixerbia* (0%, 100%)], increasing the roundness does increase the size due to how the neural network has positioned such pollen in the multidimensional latent space. It is evident that increasing the roundness does not merely make the pollen rounder, but changes the ornamentation as well. For example in **Figure 8B**, an increase in the roundness of *Carpodetus* removes the lobe structure as it transitions to *Citrus*. The conclusions drawn from these figures may be limited to the training dataset, and hence, additional training data may provide additional insights. If this approach was applied to a much more varied dataset, perhaps containing thousands of taxa, latent w-space vector arithmetic could have the potential to help understand the relationship between features and traits in pollen grains and to predict taxon changes (and results of these changes) due to environmental and evolutionary factors.

The ability to essentially upload an image into latent space for manipulation could provide a powerful tool for understanding the relationships and behaviour of pollen. In **Figure 9**, which shows the transition between two pairs of pollen images not present in training, we can see a transition between one taxon to another, not only by straight pixel interpolation but also through different taxa. In **Figure 9A**, the image transition occurs through seven different taxa, and in **Figure 9B**, the transition occurs through six different taxa, as classified by the CNN. The transition between pollen is dependent on how latent space has distributed the data, as in **Figure 9A**, the transition is not simply a large triangular shape to a small triangular shape. Although pollen grain taxa have been identified, the identification is limited to the training of 46 taxa, meaning that although a pollen grain may be identified as *Laurelia* at 99%, it may not necessarily look like it due to the limited data used in the CNN training. Owing to such as vast number of pollen taxa present in the world, being able to train a latent space neural network on every type would be extremely difficult. As such, this methodology demonstrates the possibility of using pollen taxa not used in training so that they could be explored in latent space and manipulated to understand their morphology in different environments, such as undergoing dehydration (Grant-Jacob et al., 2022a), or understand the pollens in the context of their phylogenetic relationships. This methodology could be used in addition to work on using CNNs to analyse pollen morphology and place extinct pollen morphotypes within a phylogenetic framework using Bayesian inference (Adaïmé et al., 2024).

A key challenge in isolating specific features, such as size versus shape, through w-space manipulation in StyleGAN is the potential overlap between latent vector representations for different characteristics. The latent space in StyleGAN is highly compressed and abstract, meaning that features like size, shape, ornamentation, and colour are not always entirely independent. As a result, adjusting one feature may inadvertently affect others, complicating the process of isolating and controlling a specific characteristic independently.

For example, when manipulating the latent vector to adjust the size of a pollen grain, the shape or ornamentation of the grain may also change. This is because these features may share latent dimensions in the vector space, and the model may not perfectly separate them. As seen in **Figure 7**, when the “size” vector is altered, the taxa of the pollen grain can change, along with its size, indicating a correlation between size and taxa or other features like ornamentation. This overlap in latent vector representations makes it difficult to manipulate one feature without influencing others.

Furthermore, the model’s ability to separate features effectively depends on the richness and diversity of the training dataset. If the dataset lacks sufficient variation in certain attributes, the model may struggle to create distinct latent representations for each feature. This could lead to challenges in fine-tuning or generating high-quality, realistic images where individual characteristics are clearly separated, as seen in the generated images where manipulating the “spike” vector also influenced the size and ornamentation of the pollen. These issues highlight the complexity of manipulating specific features in StyleGAN and the need for a more refined approach to disentangling latent representations.

Synthetic images generated by StyleGAN can be highly valuable for augmenting real-world datasets, especially in areas like pollen classification, where obtaining a diverse and high-quality dataset may be challenging. By generating realistic and controlled synthetic images, StyleGAN can help fill gaps in datasets, increase their size, and improve the diversity of training examples available for deep learning models. This can enhance the robustness and generalisation ability of classifiers, making them more effective in real-world applications.

For example, in the context of pollen classification, obtaining images of all possible pollen types with varying characteristics (e.g., size, shape, and ornamentation) under different imaging conditions can be difficult. Augmentation of images could be achieved by carefully manipulating latent vectors, and specific features such as spike density, size, and ornamentation can be adjusted, enabling the generation of images for underrepresented or difficult-to-capture species or scenarios. These synthetic data can thus act as a supplement to the real dataset, improving classifier performance on less common or poorly represented pollen types.

Moreover, synthetic images can be particularly useful in cases where real-world data are scarce due to privacy concerns, cost, or limited access to expert annotation. For example, in clinical or environmental settings where data collection is expensive or time-consuming, synthetic images can fill in the gaps, allowing deep learning models to be trained on a more diverse set of examples. Additionally, StyleGAN-generated images could be used to simulate edge cases or rare occurrences that may not be adequately captured in real-world datasets, further enhancing the model’s ability to handle a wide range of real-world conditions.

## 5 Conclusion and future scope

The results presented in this work demonstrate the significant potential of leveraging StyleGAN’s latent space manipulation to explore and understand pollen grain morphology. Through the projection, generation, and manipulation of synthetic pollen images, we have shown that adjusting latent vectors such as “size”, “spike”, and “roundness” not only alters the appearance of pollen grains but can also lead to changes in their taxon classification. These findings suggest that latent space manipulation offers a powerful method for studying the relationships between different features of pollen grains, such as size, ornamentation, and shape, which are crucial for understanding both environmental and evolutionary influences on pollen morphology.

However, this approach is not without limitations. The results observed in this study are inherently tied to the training dataset, which restricts the generalisability of the findings. The manipulation of latent vectors is influenced by how the data are distributed in the multidimensional latent space, and this may result in unexpected transitions, particularly when working with pollen taxa not included in the training set. Further research into expanding the training dataset to include a broader range of pollen taxa, and perhaps even thousands of species, could reveal deeper insights into the underlying relationships between pollen traits and environmental factors.

The future scope of this work involves expanding the training dataset to include a broader range of pollen taxa, which would improve the accuracy and reliability of generated images and enable more precise feature manipulation. A larger, more diverse dataset would offer deeper insights into the relationships between pollen traits and environmental factors. Additionally, the ability to manipulate pollen images in latent space could be utilised to study the effects of environmental influences, such as dehydration or climate change, on pollen morphology, and to simulate evolutionary changes in pollen structures. This approach could also be integrated with phylogenetic frameworks to better understand the evolutionary relationships between different pollen taxa, as seen in work that places extinct pollen morphotypes within a phylogenetic context. Furthermore, insights gained from latent space manipulation could enhance predictive models for pollen identification and classification, particularly for taxa not included in the training dataset. Ultimately, with continued refinement and expansion, this methodology holds the potential to improve our understanding of pollen grain morphology and its implications in areas such as agriculture, climate science, and botany.

## Data Availability

The original dataset used in training the neural network in the current study is available in the https://doi.org/10.6084/m9.figshare.12370307.v1 repository. The datasets generated and/or analysed during the current study are available at https://doi.org/10.5258/SOTON/D3108.
